# Analysis of Prevention and Treatment of Anastomotic Leakage after Sphincter-Preserving Surgery for Middle- and Low-Grade Rectal Cancer under Laparoscopy

**DOI:** 10.1155/2022/6231880

**Published:** 2022-12-07

**Authors:** Jia-He Yu, Xiang-Wu Huang, Yu-Cheng Song, Hui-Zhong Lin, Feng-Wu Zheng

**Affiliations:** ^1^The School of Clinical Medicine, Fujian Medical University, Fuzhou 350122, China; ^2^Department of Anorectal Surgery, The Affiliated Hospital of Putian University, No. 999 Dongzhen East Road, Licheng District, Putian 351100, China

## Abstract

**Background:**

Anastomotic leakage is one of the most serious complications that can occur after laparoscopic-assistedsphincter-preserving surgery for middle- and low-grade rectal cancer.

**Objectives:**

To explore the cause, prevention, and treatment of anastomotic leakage after sphincter-preserving surgery for middle- and low-grade rectal cancer under laparoscopy.

**Methods:**

The clinical data from patients with mid- and low-grade rectal cancer who underwent laparoscopic-assistedanus-preserving surgery in the anorectal surgery department of our hospital have been analyzed. Patients with a definite diagnosis, indications for laparoscopic surgery, and sphincter-preserving surgery were included in the analysis, and patients with a protective loop ileostomy and laparotomy were excluded.

**Results:**

Among the 126 patients with middle- and low-grade rectal cancer undergoing sphincter-preserving surgery under laparoscopy. There were 75 male patients and 51 female patients, ranging in age from 37 to 89 years old, with an average age of 60.2 ± 6.7. The distance from the lower edge of the rectal tumor to the anal edge was ≤10 cm. 6 developed anastomotic leakage after the operation (leakage rate of 4.7%). Moreover, turbid purulent fluid was drained from the abdominal drainage tube in three patients on the third and fourth days after the operation, and the abdominal drainage tube drained serous drainage in three more patients on the fifth and sixth days, with signs of peritonitis appearing locally. All patients received continuous flushing and negative pressure drainage with a self-made double cannula and symptomatic treatment, and all were cured and discharged.

**Conclusion:**

Many factors can cause anastomotic leakage after this operation, and adequate perioperative preparation, meticulous operation during surgery, and careful postoperative management are key factors in preventing it.

## 1. Introduction

Compared with Westerners, the prevalence of rectal cancer in Chinese people has the following characteristics. Its incidence is higher than that of colon cancer, accounting for approximately 60%, and the proportion of middle- and low-grade rectal cancer is high, accounting for approximately 60%–70% of rectal cancer cases. Most such cancers can be touched during digital rectal examination [1]. Anastomotic leakage is one of the most serious complications that can occur after laparoscopic-assistedsphincter-preserving surgery for middle- and low-grade rectal cancer, with an incidence of 3%–21% after anterior resection for rectal cancer (Dixon) [2, 3]. The main issue with anastomotic leakage is colonic content corrosion of pelvic and sacral tissues, exposed lymphoid tissues, and blood vessels. If treatment is not timely sought, the leakage will seriously affect patients' postoperative recovery and subsequent treatment and may even lead to death. This complication has a high mortality [4]. Therefore, preventing and effectively dealing with anastomotic leakage are of great significance. The clinical data from patients with mid- and low-grade rectal cancer who underwent laparoscopic-assistedanus-preserving surgery in the anorectal surgery department of our hospital have been analyzed.

## 2. Methods

### 2.1. General Information

The data of 126 patients with middle- and low-grade rectal cancer who underwent laparoscopic-assistedsphincter-preserving surgery in the anorectal surgery department of our hospital from June 2017 to December 2020 were retrospectively analyzed. An electronic colonoscopy was performed before the operation for all patients, and rectal cancer was diagnosed via pathology. This study was conducted in accordance with the declaration of Helsinki and approved by the Ethics Committee of the Affiliated Hospital of Putian University. Written informed consent was obtained from all participants.

### 2.2. Tumor Characteristics

Preoperative digital examination of the anus, computed tomography (CT), magnetic resonance imaging, and intraoperative exploration were employed in combination. Clinical tumor classification: There were 74 cases of ulcerative tumors, 32 cases of protruding tumors, and 20 cases of infiltrative tumors. Pathological classification: There were 56 cases of well-differentiated adenocarcinomas, 45 cases of moderately differentiated adenocarcinomas, and 25 cases of poorly differentiated adenocarcinomas. Clinicopathological staging (the tumor-node-metastasis (TNM) staging according to the eighth edition of the Union for International Cancer Control developed in 2017) [5]: There were 34 cases at Stage I, 69 cases at Stage II, and 23 cases at Stage III. The distance from the lower edge of the anastomosis to the anal edge after the anastomosis was >2.5 cm in 51 cases and >4 cm in 75 cases.

### 2.3. Inclusion Criteria

All patients were informed of the operation plan. The perioperative preparation was adequate, and no patients had operative contraindications. Laparoscopic-assisted radical resection for middle- and low-grade rectal cancer (Dixon) was performed for all patients. All patients were anastomosed with double staplers [6]. Patients with a protective loop ileostomy and a laparotomy were excluded. The operative procedures were approved by the hospital's ethics committee.

### 2.4. Methods

#### 2.4.1. Operation Steps

**Figure 1:**
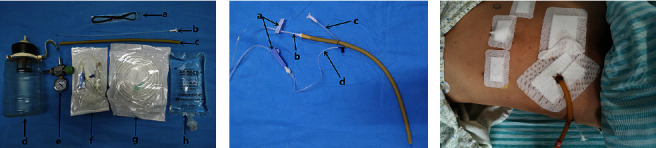
Self-made double cannula. (a) Self-made double cannula subassembly. Notes. (A) #7 silk thread, (B) #12 ventricular drainage tube, (C) rubber drainage tube, (D) negative pressure drainage bottle, (E) pressure gauge, (F) infusion set, (G) suction tube, (H) normal saline. (b) Self-made double cannula sample. Notes. (A) controllable switch, (B) connected to drainage bag, (C) connected to negative pressure suction tube, (D) flushing tube. (c) Practical application of self-made double cannula.

**Figure 2:**
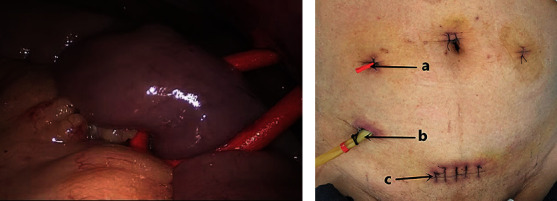
Ghost ileostomy. (a) The distance from the suspended ileum to the ileocecal part is approximately 40 cm. (b) Postoperative abdominal wall appearance. Notes. (A) fixation site at abdominal wall skin for ghost ileostomy suspension, (B) self-made double cannula, (C) median transverse incision of lower abdomen.

Each patient was placed in the modified lithotomy position [7]. Under general anesthesia, after disinfection and surgical drape placement, laparoscopic intraoperative exploration was carried out. After the relationship between the location of the rectal tumor and surrounding organs was basically determined and it was clear that the tumor had not obviously infiltrated or metastasized in the abdominal cavity, the uterus of female patients was suspended with purse strings, and an incision was made on the retroperitoneum at the junction of the sigmoid mesocolon and small pelvic cavity with an ultrasonic scalpel. The inferior mesenteric artery and veins were carefully isolated, clamped, and cut with a ligation latch clip. The adipose lymphoid tissues around the root of the inferior mesenteric artery and veins were cleaned, and the operation reached the Toldt space to the left to free the sigmoid colon. Following the total mesorectal excision (TME) and nontouch isolation principles, the lymphatic and adipose tissues near the iliac vessels and lateral pelvic wall in the presacral space between the wall and visceral layers of the pelvic fascia were incisively dissected, separated, and removed. An incision of the pelvic floor peritoneum in the rectal bladder or rectal uterine depression was made, and the rectal wall was exposed, approximately 3–5 cm away from the lower edge of the tumor. If necessary, a digital rectal examination was performed during the operation to locate the lower edge of the tumor. The intestinal canal at the anastomosis was scheduled and clamped with intestinal forceps. The anus was fully expanded, and the lower rectum was flushed with 250 ml of diluted iodophor and normal saline through the anus to avoid tumor cell contamination in the closed line as far as possible. Then, the distal end of the rectum was closed with a cutting closure device, the rectum was amputated, and the sigmoid mesosigmoid, and blood vessels were arranged. The incision of the median one-trocar hole of the lower abdomen was extended 4-5 cm along the transverse line. The severed rectum and its mesosigmoid were removed from the abdominal cavity, and the sigmoid colon was cut 10 cm from the upper edge of the tumor. The rectal tumor, sigmoid mesosigmoid and its lymphoid cells, and adipose tissue were removed. The anvil head of the tubular circular stapler, with a diameter of 29–33 mm, was placed and fixed at the stump of the sigmoid colon. The abdominal incision was sutured, and the peritoneum was rebuilt. The stapler handle was inserted into the anus and connected with the anvil head in the abdominal cavity. After confirming that there was no intestinal torsion and that no other tissue was sandwiched in the junction, the anastomosis was started. The cutting ring in the stapler was checked and sent to the doctor for pathological analysis. After the anastomosis, air was injected into the rectal cavity to check for air leakage, and a self-made double cannula [8] was placed behind and below the presacral anastomosis (Figure 1), led out through the one-trocar hole in the right lower abdomen, and fixed and connected to the external drainage bag. At the end of the operation, the pelvic cavity was rinsed with warm normal saline. If the patient had a chronic disease or weak physique, was elderly, or was unsatisfied with the anastomotic stoma, terminal ileum prophylactic suspension plus abdominal wall skin fixation with an external suspension wire was performed, and a rubber tube was placed in the anal canal to reduce pressure. A ghost ileostomy (Figure 2) refers to that at the ileum about 40 cm away from the ileocecal valve. A #12 rubber catheter passes through the ileal mesentery, and prophylactic suspension of the terminal ileum is performed. The suspended terminal ileum remains in the abdominal cavity and is not fixed to the surrounding tissue. The suspended rubber catheter is led out from the right upper abdominal one-trocar hole and fixed with external abdominal wall skin (with appropriate tightness) to ensure that the intestines are free of torsion and that the blood supply is good. If there is no obvious anastomotic leakage approximately six days after the operation, the rubber catheter can be removed. In the case of anastomotic leakage, the skin of the abdominal wall at which the rubber catheter is fixed is cut into the abdominal cavity according to the layer of the leakage, and the preventively suspended terminal ileum is removed for a colostomy.

#### 2.4.2. Specific Countermeasures for Preventing Anastomotic Leakage in Clinic

(A) Prophylactic end ileostomy or transverse colostomy is performed during the operation to divert feces. This method is suitable for patients with preoperative neoadjuvant chemoradiotherapy, advanced-age patients with chronic diseases, patients with many underlying diseases and a weak physique, patients with anastomosis tension, patients with sphincter-preservingultra-low rectal cancer, and patients undergoing emergency operation due to obstruction. (B) A postoperative end ileostomy or transverse colostomy is performed during the operation to divert feces. This method can be applied in the following situations: (1) No prophylactic measures have been taken, and anastomotic leakage occurs after the operation; after active and conservative treatment, the symptoms and signs are not improved, but the patient needs open flushing and drainage, followed by an enterostomy. (2) Prophylactic measures have been taken, and the patient has either undergone a ghost ileostomy or a self-made double cannula has been placed behind the external terminal ileum and presacral anastomosis. After the operation, anastomotic leakage that seems to be not limited occurs. Thus, direct ileostomy and double cannula continuous negative pressure flushing and drainage are performed, and active symptomatic treatment is given. (C) For limited anastomotic leakage, double cannula continuous flushing and negative pressure drainage can be performed immediately, and symptomatic treatment, such as anti-infection therapy, fasting, octreotide inhibition of digestive juice secretion, nutritional support, and rehydration, can be strengthened [9, 10]. (D) If the anastomosis is not satisfactory, double cannula continuous flushing and negative pressure drainage should be performed immediately after the operation to keep the pelvic floor clean, which is conducive to the healing of the anastomosis and reduces the occurrence of anastomotic leakage.

#### 2.4.3. The Following Precautions Should be Taken [11]

(A) Closely observe whether the self-made double cannula drainage is unobstructed or compressed, and whether there is distortion or falling off. If a blockage is found, sterile normal saline can be extracted from the syringe for repeated flushing; in the case of compression and distortion, the position should be adjusted, and the tube must be replaced so that it does not fall off. During the operation, the double cannula must be placed at the lowest position behind the anastomosis to prevent retention of effusion. Effectively control the negative pressure at ≤0.02 MPa to avoid tissue damage or tube blockage in the pelvic cavity due to excessive negative pressure. (B) The rate of continuous flushing fluid should be continuously adjusted as the drainage volume and nature of the drainage fluid change; the entire operation process must be sterile to prevent exogenous infection. (C) Observe whether the negative pressure introducer is too large or too small and whether the inlet and outlet fluid at either end of the drainage tube are balanced. (D) After continuous flushing and drainage for 4–10 days, if the drainage fluid volume decreases daily and the color of the flushing fluid becomes lighter, the double cannula should be gradually withdrawn until it is completely removed.

#### 2.4.4. Observation Indexes

The occurrence and clinical manifestations of anastomotic leakage after laparoscopic-assistedsphincter-preserving surgery for middle- and low-grade rectal cancer in 126 patients were studied and summarized, and the treatment countermeasures and effects were analyzed.

## 3. Results

Among the 126 patients with middle- and low-grade rectal cancer undergoing sphincter-preserving surgery under laparoscopy, the distance from the lower edge of the rectal tumor to the anal edge was ≤10 cm. In 81 cases (64.28%), the distance was ≥6.0–10 cm, and in 45 cases (35.72%), the distance was ≥4.5–6.0 cm, 6 developed anastomotic leakage after the operation (leakage rate of 4.7%), comprising 4 male patients and 2 female patients ranging from 35 to 88 years old. On the second and fourth days after the operation, turbid purulent fluid was drained from the abdominal drainage tube in three cases, and on the fifth and sixth days, the abdominal drainage tube drained light yellow liquid in another three cases, with abdominal distension, low fever, and local peritonitis in the lower abdomen. The anastomotic leakage was diagnosed by CT and digital anal examination. These six patients received continuous flushing and negative pressure drainage with a self-made double cannula and strengthened symptomatic treatment, such as anti-infection therapy. All patients were cured and discharged. The healing time was 4–10 days, with an average of 5.69 days. The continuous flushing time was 3–10 days, and the hospital stay length ranged from 8 to 24 days [12]. Regarding the patients with abdominal distension, low fever, and local peritonitis in the lower abdomen, their body temperature returned to normal 3-4 days after continuous flushing with a double cannula. Their abdominal distension and pain improved significantly 5–8 days after the flushing; the fluid drainage decreased, and the color of the flushing fluid became lighter after 9–15 days. At this time, the double cannula was gradually withdrawn until it was completely removed; this operation went smoothly in all patients.

## 4. Discussion

In recent years, with the continuous improvement of people's living standards and quality of life and the maturity of endoscopic technology combined with the wide clinical application of staplers, cutting closures, ultrasonic scalpels, and other instruments, patients with middle- and low-grade rectal cancer have demonstrated a significantly increased willingness to require preservation of the sphincter during surgery. Thus, clinicians have adopted laparoscopic-assisted radical resection for middle- and low-grade rectal cancer (Dixon), which makes the sphincter-preserving surgery simple and convenient. Anastomosis is a reliable operation that has the advantages of less trauma, a shorter operation time, and rapid postoperative recovery, improving patients' quality of life and the sphincter retention rate. This surgical method has also greatly promoted the technological progress of the surgical treatment of rectal cancer. However, there are still complications, such as postoperative anastomotic leakage and bleeding. In this study, the data of 126 patients with middle- and low-grade rectal cancer who underwent the sphincter-preserving surgery under laparoscopy were analyzed and studied. Of these patients, six developed anastomotic leakage after the operation. They received continuous flushing with normal saline or continuous washing with normal saline and dripping metronidazole alternately, negative pressure drainage with a self-made double cannula, and strengthened symptomatic treatment, such as anti-infection therapy. All six patients were cured and discharged. This suggests that continuous flushing and negative pressure drainage with a self-made double cannula play an obvious role in preventing and treating anastomotic leakage simply, economically, and effectively.

The clinical study analysis also revealed that the results of timely, effective, and active conservative treatment of anastomotic leakage should be significantly better than those of surgical treatment. Most anastomotic fistulas heal 1–3 weeks after conservative treatment [13].

In summary, patients with middle- and low-grade rectal cancer should be fully evaluated before operation to recognize the risk factors for anastomotic leakage. Therefore, adequate perioperative preparation, meticulous operation during surgery, and careful postoperative management are key factors in preventing anastomotic leakage. In the case of such leakage, thorough continuous pelvic flushing and effective pelvic drainage are vital to reducing and controlling abdominal infection and promoting healing of the leakage. It is only in this way that we can ensure the most ideal operation results.

## Data Availability

The data used to support the findings of this study are available from the corresponding author upon request.
